# Host drivers of canine dirofilariosis in an arid environment of western Argentina

**DOI:** 10.1007/s00436-024-08367-y

**Published:** 2024-10-09

**Authors:** Pablo Fernando Cuervo, Sophia Di Cataldo, María Cecilia Fantozzi, María Belén Rodríguez, Analía Pedrosa, Roberto Mera y Sierra

**Affiliations:** 1 Centro de Investigación en Parasitología Regional, Universidad Juan Agustín Maza, Guaymallén, Mendoza Argentina; 2 Departamento de Parasitologia, Facultad de Farmacia, Universidad de Valencia, Av. Vicent Andres Estelles s/n, Burjassot, 46100 Valencia, Spain; 3 CIBER de Enfermedades Infecciosas, Instituto de Salud Carlos IIII, C/ Monforte de Lemos 3-5. Pabellón 11. Planta 0, 28029 Madrid, Spain; 4 Instituto de Medicina y Biología Experimental de Cuyo (IMBECU), Consejo Nacional de Investigaciones Científicas y Tecnológicas (CONICET), Mendoza, Argentina; 5 Laboratorio de Enfermedades Zoonóticas y Vectoriales, Ministerio de Salud de Mendoza, Mendoza, Argentina; 6 Histología y Embriología Veterinaria / Laboratorio de Genética, Ambiente y Reproducción, Facultad de Ciencias Veterinarias y Ambientales, Universidad Juan Agustín Maza, Guaymallén, Mendoza Argentina

**Keywords:** *Dirofilaria immitis*, Heartworm disease, Risk factors, Odds ratio

## Abstract

Canine dirofilariosis is a mosquito-borne zoonotic disease largely caused by *Dirofilaria immitis*. Mainly present in temperate, semitropical, and tropical areas, its worldwide emergence and spread are causing concern. In Argentina, most cases have been reported in humid regions of the center and northeast of the country. The occurrence of canine dirofilariosis in an arid environment of western Argentina was investigated by blood smears, microhematocrit tube test, and Knott’s technique. Association and odds ratio were determined in relation to dog characteristics. Thirty-three dogs (51.6%) had microfilariae, morphologically identified as *D. immitis*. Knott’s test was more sensitive in detecting the positive animals. The odds of harboring microfilariae were 12, 29, and 66 if the dog was male, adult (three to 6 years old), or older (> 7 years old), respectively. The prevalence of canine dirofilariosis herein reported outstands among the highest in Argentina and extends to the west its geographic distribution in the country. Far from being an exception, this epidemiological situation might reflect similar circumstances in several arid locations in the west and center of Argentina. Being this a disease of recent appearance in the region, both veterinarians and physicians should be aware of its potential to cause disease in animals and humans.

**Supplementary Information:**

The online version contains supplementary material available at 10.1007/s00436-024-08367-y.

## Introduction

Canine dirofilariosis is a mosquito-borne zoonotic disease mainly caused by the heartworm *Dirofilaria immitis* (Simón et al. 2012). In dogs, considered the main reservoirs, the parasite causes cardiopulmonary disease, while humans are considered “dead-end” hosts, sporadically presenting benign pulmonary nodules. The worldwide spread of *Dirofilaria* spp. is causing concern, as emergence foci are being reported in several countries, mainly attributed to climate and global changes (Genchi et al. 2011; Perles et al. 2024).

As the life cycle of mosquitoes is itself linked to water, the distribution of *Dirofilaria* spp. is strongly influenced by climatic factors, being present in temperate, semitropical, and tropical areas worldwide (Simón et al. 2012; Perles et al. 2024). Indeed, in Argentina, canine dirofilariosis has been reported mainly in temperate and humid regions of the center and northeast of the country (reviewed in Vezzani et al. 2006 and Vezzani and Eiras 2016). However, its presence has been sporadically reported in other provinces, such as Mendoza (Cuervo et al. 2013b) and La Pampa (Uhart et al. 2012), where climatic conditions were presumed to be less suitable, mainly due to scarce precipitation. In fact, a local communication suggested the existence of an unexpected transmission cycle of *Dirofilaria* spp. in an arid area of northern Mendoza (Pedrosa et al. 2012).

In view of the threat to domestic dogs and the public health risk, we aimed (i) to confirm the occurrence of *Dirofilaria* spp. in dogs from an arid location in western Argentina; (ii) to identify the species of *Dirofilaria* present; and (iii) to recognize risk factors related to the host.

## Methods

**Fig. 1:**
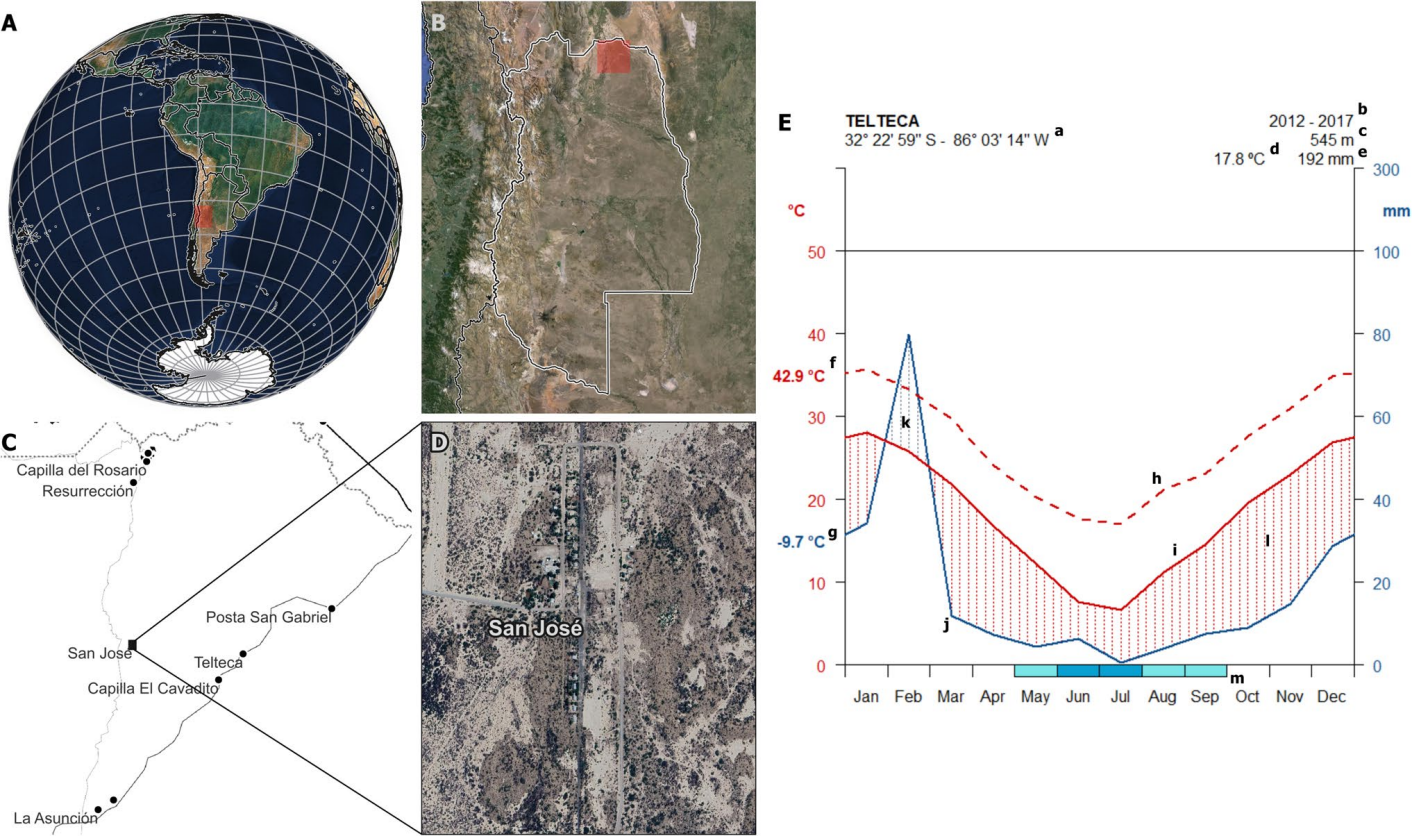
Geographical location of San Jose (**A**–**D**) and climate diagram (**E**), showing its extreme environmental conditions. Climate diagram constructed based on original meteorological data from the nearby Telteca weather station (IADIZA, CCT CONICET Mendoza, https://www.mendoza-conicet.gob.ar/ladyot/red_iadiza/index.htm, accessed on 15 April 2024). References: (a) geographical coordinates; (b) time period analyzed; (c) altitude, in meters above sea level; (d) mean annual temperature; (e) mean annual precipitation; (f) mean annual maximum temperature; (g) mean annual minimum temperature; (h) maximum temperature; (i) mean temperature; (j) minimum temperature; (k) wet period; (l) arid period; (m) frost period

The study took place in November 2013 and included every domestic dog living at least during the last 3 months in scattered rural outposts from the locality of San Jose (32° 21′ 59.64″ S—68° 13′ 3.37″ W), in Mendoza province (Fig. 1). San Jose lies amid the one-time exuberant Guanacache wetland, which has been slowly dried up during the last two centuries, resulting in a profound desertification process of the area (Escolar and Saldi 2017). Currently, this rural district is majorly covered by non-irrigated dryland, where extensive goat farming is common as the main economic activity for subsistence (Abraham and Torres 2014; Cuervo et al. 2013c). Mean annual maximum and minimum temperatures are 26 °C and 9 °C, respectively, with summer temperatures of up to 43 °C (Fig. 1). Summer rains constitute about half the average yearly precipitation of 190 mm.

Whole blood samples from 64 dogs were taken from the cephalic vein, collected in tubes with EDTA, and stored at 4 °C for microfilariae examination within 24 h. Sampled dogs were categorized considering sex, breed (pure breed, crossbreed), age (< 1 year; 1–2 years; 3–6 years; and older than 7 years), size (small, medium, and big), hair length (short or long), and body condition (cachectic 1; slim 2; medium 3; fat 4; obese 5) (details are presented as Supplementary information (SI)).

We assessed the presence of microfilariae by (i) microscopic examination of Giemsa-stained slides; (ii) microhematocrit tube technique; and (iii) modified Knott technique. After centrifugation for 5 min at 10,000 × *g*, the microhematocrit tube technique allows for the concentration of circulating microfilariae from a small amount of blood (80 μL) in the buffy coat interface (Collins 1971). Although filarial species identification is uncertain by this procedure, it permits to detect the characteristic movement pattern of microfilariae. The modified Knott technique was performed as described in Genchi et al. (2007) and typical morphology of microfilariae was observed to identify the species involved (Magnis et al. 2013; Gruntmeir et al. 2023).

We employed generalized linear models (GLMs) with binomial error distribution and log link function to analyze the relationship between the presence of microfilaremia in domestic dogs and our set of host factors (sex, breed, age, size, hair length, and body condition). We considered the complete model set that included all possible combinations of predictor variables (a total of 64 models). Interactions among predictor variables were not tested because we could not specify a priori how the effects of any covariate would vary across values of any of the other. We fitted GLMs via maximum likelihood and evaluated their relative performance with Akaike’s information criterion corrected for small sample size (AICc) (Burnham and Anderson 2002). Based on the AICc values, we selected the top-ranking candidate models that summed 0.9 cumulative AICc weight (*w*_*i*_). Parameter estimates for predictor variables were averaged based on *w*_*i*_ from all candidate models. To determine evidence of relevant effects, we determined 95% confidence intervals (CI) of parameter estimates, where those terms which did not include zero in their CI are considered different from zero and thus of importance.

Statistical analyses were carried out using R software, Version 4.2.2 (R Core Team 2022).

## Results and discussion

The presence of microfilariae was diagnosed in 34 dogs (51.6%, CI 40.65–62.55): (i) four (6.25%) were diagnosed by microscopic examination; (ii) 20 (31.3%) by the microhematocrit tube technique; and (iii) 33 (51.6%) by the modified Knott technique (Fig. S1 in SI). The very low diagnostic power of the microscopic examination of the Giemsa-stained slides dismisses its utility as a reliable diagnostic method and was excluded from further comparison. From the other two, results obtained with the modified Knott technique proved to be greater (proportion test, *p* = 0.03), detecting 1.6 times more cases than the microhematocrit tube technique. Thus, the modified Knott technique stands as a sound low-cost diagnostic method to be used in exploratory field studies. After the detection of cases in a given area, it should be complemented with more sensitive diagnostic methods (i.e., serologic and molecular techniques) to describe the epidemiological situation. These complementary methods could not be performed in this study due to their higher costs and more complex implementation.

**Fig. 2:**
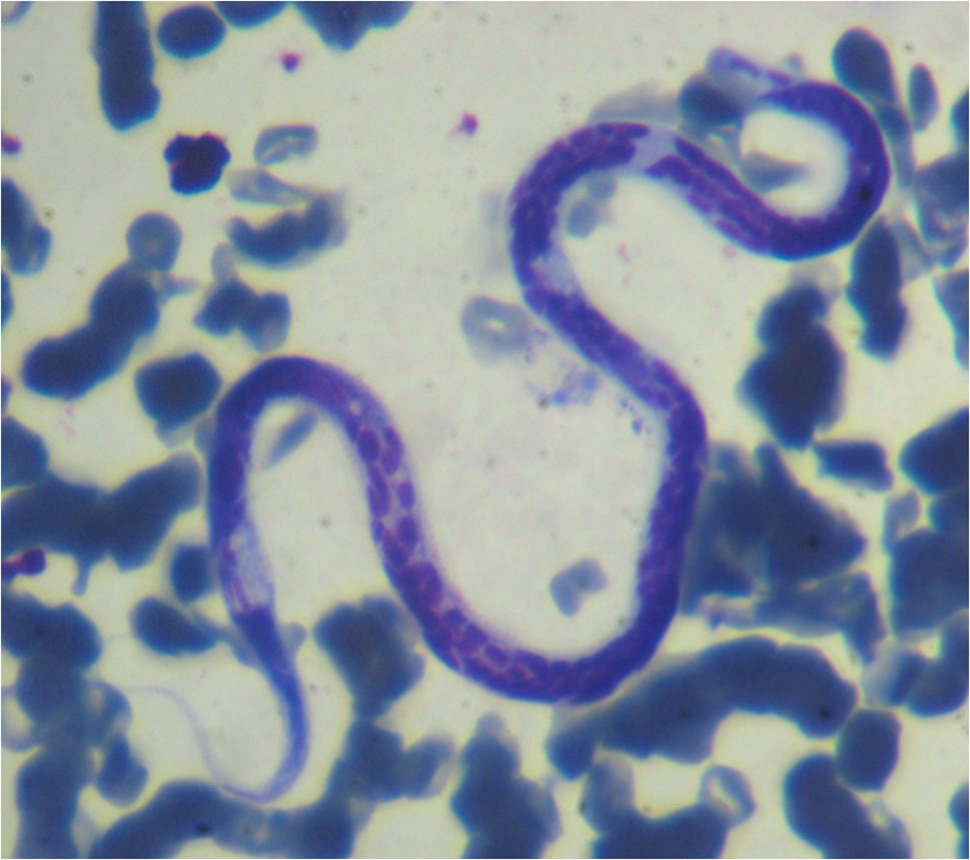
Microscopic observation of a *Dirofilaria immitis* microfilaria identified in a Giemsa-stained thin blood smear

All microfilariae detected were identified as *Dirofilaria immitis* based on their morphological characteristics (size, absence of sheath and cephalic hook, pointed cephalic and caudal ends, and tail straight [see Genchi et al. 2007; Magnis et al. 2013]) (Fig. 2). Morphological analyses have proven to be a very useful, quick, and inexpensive diagnostic tool and represent the first step in the diagnosis of filarial infections (Magnis et al. 2013), which is the case. However, caution should be exercised as microfilariae species might get confused (i.e., Lopez et al. 2012; Cancino-Faure et al. 2024; Esteban-Mendoza et al. 2024), and molecular studies are required to reach an unambiguous identification.

**Fig. 3:**
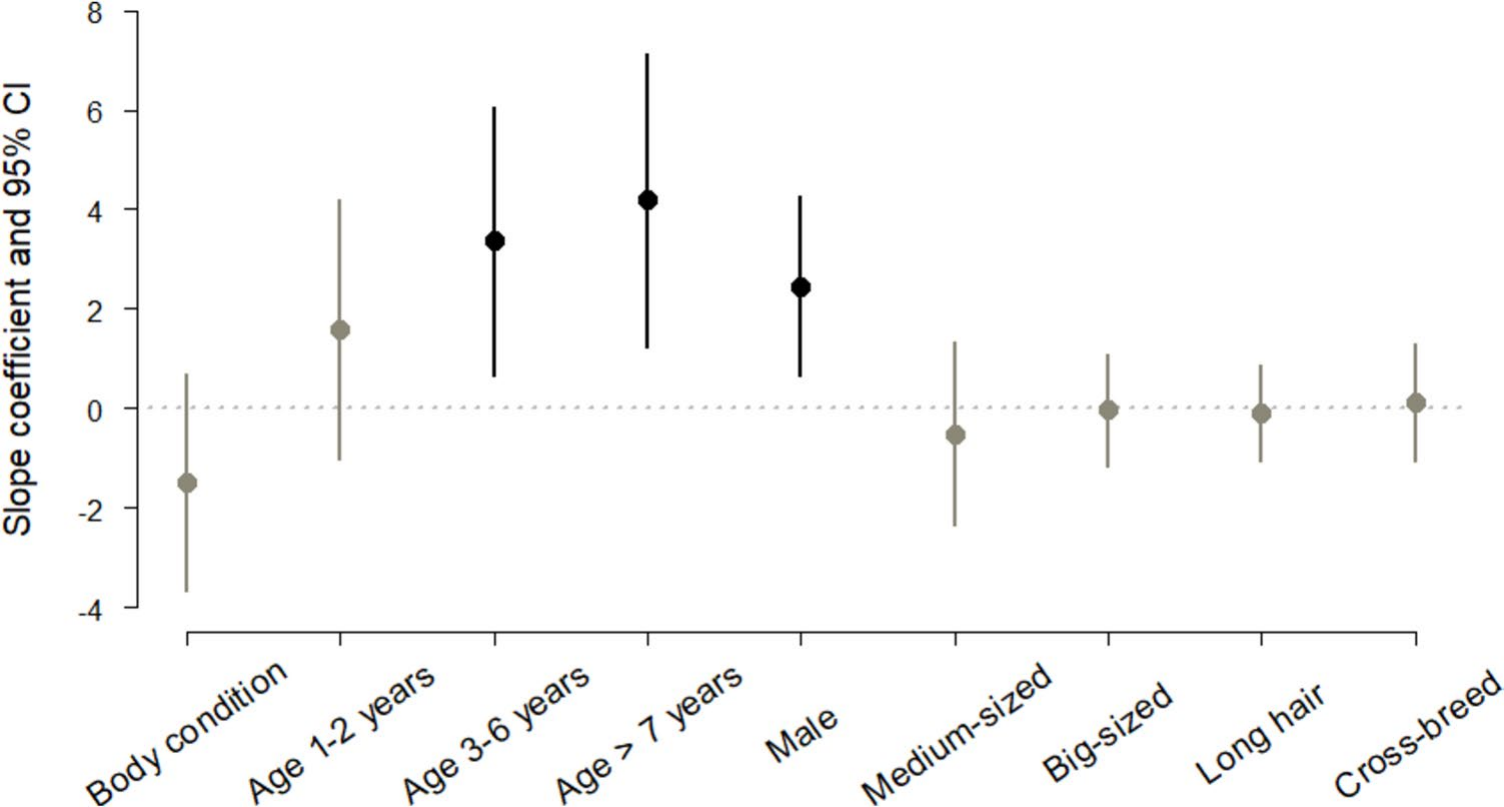
Unconditional model-averaged estimates of predictors effects on presence of microfilaremia in domestic dogs from an arid environment. Effects are considered different from zero (black symbols) when the 95% CIs do not cross the horizontal line at zero

Regarding host factors associated with the presence of microfilaremia in domestic dogs from an arid environment, the averaged results obtained from a selected subset of models (Table S1 in SI) indicate that adult male dogs are more prone to get infected (Fig. 3). While we found that the odds of male dogs presenting microfilaremia were 12 times greater than in females (Table S2 in SI), canine dirofilariosis association with sex seems to be circumstantial. Justified by the time spent outdoors, a number of studies have found that either male (Montoya et al. 1998; Rosa et al. 2002; Vezzani et al. 2011) or female dogs (Bolio-Gonzalez et al. 2007) are more predisposed to the infection with heartworm, but some others have found no difference between sexes (Montoya et al. 2006; Vieira et al. 2014).

On the other hand, coincident with previous studies (e.g., Montoya et al. 1998; Rosa et al. 2002; Bolio-Gonzalez et al. 2007; Vezzani et al. 2011; Vieira et al. 2014), heartworm infection appeared to be greater among older dogs (Fig. 3). We found that adult dogs (3–6 years old) have 29 times more odds of being microfilaremic, which increased to 66 times for dogs older than 7 years (Table S2). It is widely agreed that this might be related to a longer exposure to mosquito bites and thus greater opportunities for getting infected.

Considering that up to 30% of infected dogs might not present circulating microfilariae (McCall et al. 2008), the real prevalence of canine dirofilariosis is far beyond the minimum 40% here reported and outstands among the highest in Argentina (reviewed in Vezzani and Eiras 2016). Moreover, it considerably extends to the west the disease’s geographic distribution in the country and confirms the predictions of previous temperature-based modelling approaches (Vezzani and Carbajo 2006; Cuervo et al. 2013a, 2015). Far from being an exception, this epidemiological situation might reflect similar circumstances in several arid locations of the west and center of Argentina. As happens with other water-linked diseases, transmission concentrates in small areas where humans and animals have access to water, and where vectors dwell. Yet, veterinarians and physicians rarely suspect the occurrence of this zoonosis in the region. Since the experience in its diagnosis is scarce, the possibility of overlooked human cases is evident. Further studies are required to fully assess the progress of this neglected epidemiological situation.

## Supplementary Information

Below is the link to the electronic supplementary material.

Supplementary file1 (DOCX 84 KB)

## Data Availability

The main data is provided within the manuscript or supplementary information files. The datasets generated for this study are available upon reasonable request to the corresponding author.
