# Supplementary material for: Host drivers of canine dirofilariosis in an arid environment of western Argentina
Source: Parasitol Res. 2024 Oct 9;123(10):345. doi: 10.1007/s00436-024-08367-y (PMC11464612; doi:10.1007/s00436-024-08367-y)
Supplement: Supplementary file 1 — Supplementary file1 (DOCX 84 KB) [file 436_2024_8367_MOESM1_ESM.docx]

**PARASITOLOGY RESEARCH**

**Supplementary Information**

**Host drivers of canine dirofilariosis in an arid environment of western Argentina**

Pablo Fernando Cuervo^1,2,3^, Sophia Di Cataldo^1,4^, María Cecilia Fantozzi^1,2,3^, María Belén Rodríguez^5^, Analía Pedrosa^5,6^, Roberto Mera y Sierra^1^

1. Centro de Investigación en Parasitología Regional, Universidad Juan Agustín Maza, Guaymallén, Mendoza, Argentina.

2. Departamento de Parasitologia, Facultad de Farmacia, Universidad de Valencia, Av. Vicent Andres Estelles s/n, 46100 Burjassot, Valencia, Spain.

3. CIBER de Enfermedades Infecciosas, Instituto de Salud Carlos IIII, C/ Monforte de Lemos 3-5. Pabellón 11. Planta 0, 28029 Madrid, Spain.

4. Instituto de Medicina y Biología Experimental de Cuyo (IMBECU), Consejo Nacional de Investigaciones Científicas y Tecnológicas (CONICET), Mendoza, Argentina

5. Laboratorio de Enfermedades Zoonóticas y Vectoriales, Ministerio de Salud de Mendoza, Mendoza, Argentina.

6. Histología y Embriología Veterinaria / Laboratorio de Genética, Ambiente y Reproducción, Facultad de Ciencias Veterinarias y Ambientales, Universidad Juan Agustín Maza, Guaymallén, Mendoza, Argentina.

**Corresponding author:**

Pablo F. Cuervo (e-mail: Pablo.F.Cuervo@uv.es)

**Characteristics of the dogs sampled**

The 64 dogs sampled were categorized as following: 46 males (71.9%) and 18 females (28.1%); seven pure breed (11%) and 57 cross-breed (89%); eight with less than one year (12.5%), 17 one-two years old (26.6%), 25 three-six years old (39.1%), and 14 older than seven years (21.9%); 28 small-sized (43.8%), 23 medium-sized (35.9%), and 13 big-sized (20.3%); 54 with short hair (84.4%) and 10 with long hair (15.6%); and 17 with a medium body condition (26.6%), 24 slim (37.5%), and 23 between both categories (35.9%).


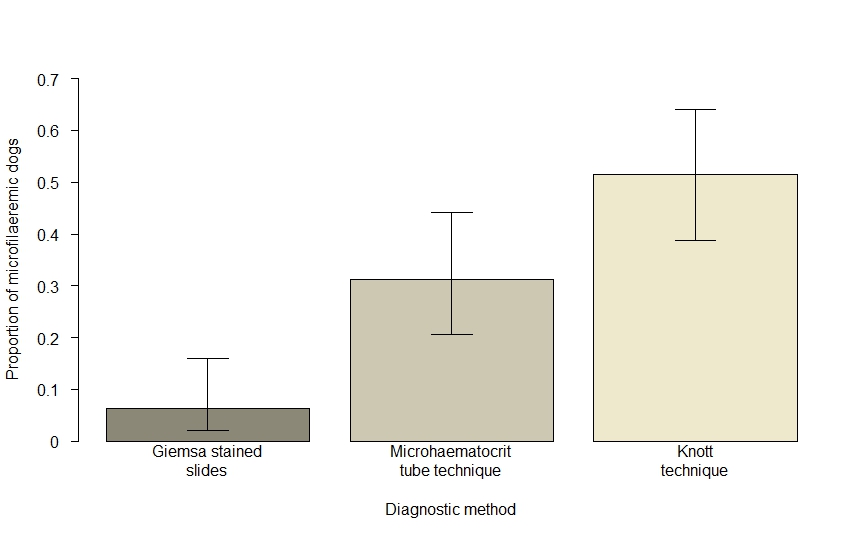


**Figure S1**. Proportion of domestic dogs diagnosed with microfilaremia, according with the diagnostic method used (whiskers indicate 95% CI).

**Supplementary Tables**

**Table S1.** Covariate structure of the top-ranking models (summed 0.9 cumulative AICc weight).

| ΔAICc | *w_i_* | Body condition | Age | Sex | Size | Hair length | Breed |
| --- | --- | --- | --- | --- | --- | --- | --- |
| 0 | 0.308 | ● | ● | ● |  |  |  |
| 1.262 | 0.164 | ● | ● | ● | ● |  |  |
| 2.150 | 0.105 |  | ● | ● |  |  |  |
| 2.211 | 0.102 | ● | ● | ● |  | ● |  |
| 2.232 | 0.101 | ● | ● | ● |  |  | ● |
| 3.298 | 0.059 |  | ● | ● | ● |  |  |
| 3.766 | 0.047 | ● | ● | ● | ● | ● |  |
| 3.877 | 0.044 | ● | ● | ● | ● |  | ● |
| 4.288 | 0.036 |  | ● | ● |  |  | ● |
| 4.521 | 0.032 | ● | ● | ● |  | ● | ● |

**Table S2.** Model-averaged estimates, odds ratios, unconditional standard errors (SE), and 95% confidence interval limits (CI_lower_, CI_upper_) from 64 models fitted for microfilaremia in dogs. The asterisk (*) signals the terms which did not include zero.

| **Predictor** | **Estimate** | **Odds Ratio** | **SE** | **CI_lower_** | **CI_upper_** |
| --- | --- | --- | --- | --- | --- |
| Intercept | -0.470 | 0.625 | 2.803 | -6.038 | 5.097 |
| Body condition | -1.486 | 0.156 | 1.101 | -3.669 | 0.697 |
| Age 1-2 years | 1.598 | 4.943 | 1.302 | -1.008 | 4.205 |
| Age 3-6 years * | 3.358 | 28.738 | 1.344 | 0.669 | 6.047 |
| Age > 7 years * | 4.194 | 66.280 | 1.474 | 1.242 | 7.146 |
| Sex: male * | 2.461 | 11.714 | 0.907 | 0.649 | 4.272 |
| Medium-sized | -0.515 | 0.194 | 0.932 | -2.356 | 1.326 |
| Big-sized | -0.040 | 0.880 | 0.557 | -1.156 | 1.075 |
| Long hair | 0.099 | 0.580 | 0.479 | -1.055 | 0.857 |
| Crossbreed | 0.119 | 1.742 | 0.592 | -1.063 | 1.300 |
